# Essential Oils and Biological Activities of *Eucalyptus falcata*, *E. sideroxylon* and *E. citriodora* Growing in Tunisia

**DOI:** 10.3390/plants12040816

**Published:** 2023-02-11

**Authors:** Ismail Amri, Marwa Khammassi, Rayda Ben Ayed, Sana Khedhri, Manel Ben Mansour, Oumayma Kochti, Ylenia Pieracci, Guido Flamini, Yassine Mabrouk, Samia Gargouri, Mohsen Hanana, Lamia Hamrouni

**Affiliations:** 1 Laboratory of Biotechnology and Nuclear Technology, National Center of Nuclear Science and Technology, Sidi Thabet, B.P. 72, Ariana 2020, Tunisia; 2 Laboratory of Management and Valorization of Forest Resources, National Institute of Researches on Rural Engineering, Water and Forests, P.B. 10, Ariana 2080, Tunisia; 3 Laboratory of Extremophile Plants, Centre of Biotechnology of Borj-Cédria, B.P. 901, Hammam-lif 2050, Tunisia; 4 Department of Agronomy and Plant Biotechnology, National Institute of Agronomy of Tunisia (INAT), University of Carthage-Tunis, 43 Avenue Charles Nicolle, El Mahrajène 1082, Tunisia; 5 Laboratory of Plant Protection, National Institut of Agronomic Research of Tunisia, P.B. 10, Ariana 2080, Tunisia; 6 Dipartimento di Farmacia, via Bonanno 6, University of Pisa, 56126 Pisa, Italy

**Keywords:** allelochemicals, biopesticides, weeds, fungi, plant interactions

## Abstract

Many plants are able to synthesize essential oils (EOs), which play key roles in defense against weeds, fungi and pests. This study aims to analyze the chemical composition and to highlight the antioxidant, antimicrobial and phytotoxic properties of the EOs from *Eucalyptus falcata*, *E. sideroxylon* and *E. citriodora* growing in Tunisia. EOs were analyzed by gas chromatography coupled to mass spectrometry (GC/MS) and their antioxidant properties were determined by total antioxidant capacity (TAC), DPPH and ABTS assays. The phytotoxic potential was assessed against weeds (*Sinapis arvensis*, *Phalaris canariensis*) and durum wheat crop (*Triticum durum*) and compared to chemical herbicide glyphosate. The antifungal activity was investigated in vitro against eight target fungal strains. All EOs displayed a specific richness in oxygenated monoterpenes (51.3–90%) and oxygenated sesquiterpenes (4.8–29.4%), and 1,8-cineole, citronellal, citronellol, *trans*-pinocarveol, globulol, spathulenol and citronellyl acetate were the main constituents. *Eucalyptus* EOs exhibited remarkable antioxidant activity and *E. citriodora* oil exhibited significant activity when compared with *E. falcata* and *E. sideroxylon* EOs. The phytotoxic potential of the tested oils had different efficacy on seed germination and the growth of seedlings and varied among tested herbs and their chemical composition variability. Their effectiveness was better than that of glyphosate. At the post-emergence stage, symptoms of chlorosis and necrosis were observed. Furthermore, a decrease in chlorophyll and relative water content, electrolyte leakage and high levels of MDA and proline were indicators of the oxidative effects of EOs and their effectiveness as bioherbicides. Moreover, all the EOs exhibited moderate fungitoxic properties against all the tested fungal strains. Therefore, according to the obtained results, *Eucalyptus* EOs could have potential application as natural pesticides.

## 1. Introduction

Chemical pesticides are among the most applied agrochemicals for pest control. However, their repeated application has resulted in an increased risk of emergence of pest resistance and soil degradation, making the overuse of these synthetic pesticides a new problem for global environment preservation [1,2]. The potential hazardous effect on the environment and on human and animal health associated with the abuse of chemicals has encouraged scientists to search for eco-friendly alternatives for pest control [3,4]. The search for alternatives to these chemical pesticides with natural-based products and biological molecules would provide a sustainable solution to protect both crops and the environment [5]. In this context, allelochemicals are molecules synthesized by plants and known to have several biological activities. These molecules are particularly involved in the defense systems of plants against pests and are also synthesized as part of the allelopathy process [6,7,8]. It has been reported that several allelochemical molecules have different biological activities. They can act as phytotoxic molecules and thus inhibit the germination and growth of several plant species [8,9]. They can exert different types of effects against insects, both attractive and repellent, and even insecticidal and larvicidal effects [3]. They are also known to inhibit the growth of various microorganisms, such as bacteria, fungi and yeasts [10,11,12].

In this way, allelochemicals could be of great interest for the development of new eco-friendly bio-pesticides [11,13]. In fact, these molecules have a short half-life and therefore problems regarding the persistence of residues in the environment can be solved and they can be considered safe and eco-friendly for the environment [13].

EOs plays a crucial role in the allelopathic interactions between plants and species of their biotopes, such as weeds, fungi, bacteria and insects [3,5,10]. Terpenes are known as potent allelochemicals with herbicidal and antifungal activity [6]. These molecules can be a good source for the discovery of natural fungicides and herbicides and can limit the harmful environmental effects caused by chemical pesticides [11]. According to the literature, the EOs of various plants have been reported to possess a significant phytotoxic effect against weeds [5,8,11]. Hence, exploiting the allelopathic potential of molecules with herbicidal properties could be a promising approach for weed control [4,7,11,14].

The *Eucalyptus* genus is part of the *Myrtaceae* family, comprising forest trees native to Australia; the *Eucalyptus* genus includes 13 subgenera and more than 700 species [15]. More than 100 species of *Eucalyptus* have been introduced in Tunisia since the 1950s. These species have been used for reforestation programs. They are mainly exploited for wood production, erosion control and the production of EOs. *Eucalyptus* species EOs are a mixture of volatile terpenes, such as 1,8-cineole, globulol, citronellol, spathunelol, limonene and pinenes [5,8,9].

Various molecules extracted from *Eucalyptus* species have been reported to have toxic effects against weeds, insects and microorganisms [5,8,9,15]. Plants’ secondary metabolites, known to possess several activities, and their applications in the field make them good alternatives for chemical pesticides without side effects—in particular, the persistence linked to the non-biodegradability of pesticides and resulting in toxicity to the environment, cultivated plants, wild animals, beneficial microorganisms and several insects [13,16].

The essential oils of *E. falcata*, *E. sideroxylon* and *E. citriodora*, which grow in Tunisia, have been the subject of recent studies [15,17,18,19]; however, studies of their herbicidal potential have not been performed. Likewise, the majority of studies on their antifungal potential are focused on clinical fungi, and few are studies of phyto-pathogenic fungi [17]. The herbicidal activity of *Eucalyptus citriodora* has been reported in India [20], but their mode of action was not described. On the other hand, knowing that chemical ecology is a very important strategy, it is widely exploited for the discovery of metabolites because plants are able to synthesize various molecules with the aim of adaptation and acclimatization to environmental pressure. The synthesis of these molecules continues to evolve according to the pedoclimatic conditions of their biotopes in order to act in a physiological or ecological way on the maintenance of the plant in its ecological environment [13]. This may explain the variability in the production of essential oils depending on the origin of the plant and the usefulness of testing plant material from different origins.

In this sense, the present work aims to determine the chemical compositions of the EOs of *E. falcata*, *E. sideroxylon* and *E. citriodora* growing in Tunisia, to evaluate their antioxidant potential and to test their antifungal effects against eight strains of phyto-pathogenic fungi, as well as to study their herbicidal activity against the germination and seedling growth of weeds and cultivated crops at the germination stage. Moreover, at the post-emergence stage, our objective is to test the effect of EOs applied by spraying on the physiological effects of plants: the relative water content, the relative leakage of electrolytes, the synthesis of chlorophyll, the rate of malondialdehyde resulting from lipid peroxidation and also the proline content, always referring to the activity of a reference synthetic herbicide: glyphosate.

## 2. Materials and Methods

### 2.1. Plant Material

*Eucalyptus* species used for essential oil extraction and weed species tested for herbicide trials, sampling origin, climatic conditions and dates of collection are listed in Table 1. For each *Eucalyptus* species, five samples were harvested from different trees at least 20 m away. *Eucalyptus* samples were then stored in a greenhouse for drying until constant weight.

Plants were identified by Professor Lamia Hamrouni, and the voucher specimens were deposited in the herbarium section of the Institute (INRGREF).

### 2.2. Extraction of Essential Oils

EOs were obtained by hydrodistillation of dried leaves in a Clevenger-type apparatus. The extraction lasted 4 h for each sample. EOs were collected and dried over anhydrous sodium sulfate and stored in a brown glass bottle at 4 °C until use. Yield was calculated based on dried weight (*w*/*w* %).

### 2.3. Gas Chromatography and Mass Spectrometry Analysis

Gas chromatography/electron ionization–mass spectrometry (GC/EI–MS) was performed using an Agilent 7890B gas chromatograph (Agilent Technologies Inc., Santa Clara, CA, USA) equipped with an Agilent HP-5MS capillary column (30 m × 0.25 mm; coating thickness 0.25 μm) and an Agilent 5977B single quadrupole mass detector. The analytical conditions were as follows: oven temperature programmed from 60 °C to 240 °C at 3 °C/min; injector temperature 220 °C; transfer-line temperature 240 °C; carrier gas helium at 1 mL/min. The acquisition parameters were as follows: full scan; scan range: 35–300 *m*/*z*; scan time: 1.0 s; threshold: 1 count. The identification of the constituents was based on the comparison of their retention times with the retention times of pure reference samples and comparing their linear retention indices (LRIs) relative to the series of n-alkanes. The mass spectra were compared with those listed in the commercial libraries NIST 14 [21] and Adams (2007) [22] and in a home-made mass-spectral library, built using MS literature combined with data experimentally obtained from pure substances.

### 2.4. Antioxidant Activity of EOs

#### 2.4.1. Total Antioxidant Capacity

The total antioxidant capacity (TAC) was estimated by an assay based on the reduction of Mo (VI) to Mo (V) by EOs and on the formation of a green phosphate/Mo (V) complex at acidic pH [23]. One hundred µL of each oil was added to 1 mL of reagent solution containing sodium phosphate (28 mM), H_2_SO_4_ (0.6 M) and ammonium molybdate (4 mM). Then, the mixtures were heated for 90 min at 95 °C, followed by cooling, and the absorbance was read at 695 nm. The antioxidant capacity was expressed as mg gallic acid equivalents per gram of EOs (mg GAE/g EOs). All tested oils were analyzed in triplicate.

#### 2.4.2. DPPH Assay

The free radical scavenging activity of *Eucalyptus* oils was assessed by the DPPH (1,1-diphenyl-2-picrylhydrazyl) radical scavenging method, according to Hanato et al. (1988) [24]. For this, 400 μL of each oil diluted with methanol at different concentrations or pure methanol (control) was mixed with 4 mL of methanolic solution of DPPH (0.1 mM). Then, the samples were vortexed and incubated for 30 min at room temperature in the dark, and the absorbance was measured at 517 nm. The percentage of DPPH scavenging activity was calculated according to the following equation:

% inhibition = (A control − A sample/A control) * 100

where A sample is the absorbance value of the tested oil and A control is the absorbance value of the control.

The IC_50_ is defined as the concentration of EOs required to scavenge 50% of the free radicals. All tested oils were analyzed in triplicate. Butylated hydroxytoluene (BHT) was used as a positive control.

#### 2.4.3. ABTS Free Radical Scavenging Activity

The ABTS+ assay was assessed following the methods described by Re et al. (1999), with slight modifications [25]. A solution containing ABTS radical cations was prepared by mixing an equal volume of potassium persulfate (2.45 mM) and ABTS (7 mM). The mixture was incubated for 16 h in the dark at room temperature. The absorbance of the ABTS solution was adjusted to 0.70 ± 0.02 at 734 nm. A 400 µL aliquot of various concentrations of *Eucalyptus* EOs or pure methanol for the control was mixed with 4 mL of the ABTS radical solution and allowed to stand at room temperature for 5 min in the dark. Absorbance was then measured at 734 nm. Trolox was used as the standard.

The percentage inhibition of the radical cation ABTS^+^ was determined using the following formula:

Inhibition of ABTS (%) = ((Ac − As)/Ac) × 100

where Ac is the absorbance of the control and As is the absorbance of the tested oil.

ABTS scavenging activity is expressed as the IC_50_ value (μg/mL). All tested oils were analyzed in triplicate.

### 2.5. Seed Germination and Seedling Growth Experiments

Seeds of two weeds, *P. canariensis* L. and *S. arvensis* L., and a cultivated species, *T. durum* L., were used in herbicidal activity assays. Before germination tests, seeds were disinfected with 5% sodium hypochlorite, and then rinsed with water. Twenty seeds were placed in Petri dishes lined with a double layer of Whatman No. 1 filter paper. They were then treated with different doses (0, 0.5, 1, 1.5, and 2 μL/mL) of *Eucalyptus* oils in a solution of Tween 20 (0.1%) and incubated for 12 days at 25 °C; glyphosate was used as a positive control and the choice of glyphosate was based on the fact that it is the active molecule of several commercial herbicides used for the control of annual and perennial grasses and broad-leaved weeds in the post-planting/pre-emergence of many crops [7,16]. The tests were carried out in a completely randomized manner with three replicates for each dose. After 12 days, germination percentages and measured root and shoot growth (cm) were calculated.

### 2.6. Post-Emergence Assays

The post-emergence application of *Eucalyptus* EOs was performed to study its herbicidal effects on 21-day-old plants under controlled conditions in a glass greenhouse. Twenty seeds of the tested plant species were sown in polypropylene pots containing 500 g of mixture of peat, sand and perlite (1/3 of each). Fifteen days after seedling emergence, only five equal-sized plants per pot were kept.

When plants were 21 days old, they were sprayed with the following different solutions at 100 mL/m^2^:

- Solution of water/Tween 20 (0.1%) as negative control;
- Solution of 10 µL/mL of *Eucalyptus* EOs dissolved in water/Tween 20 (0.1%);
- Solution of 10 mg/mL of glyphosate dissolved in water/Tween 20 (0.1%) as positive control.

A total of 15 pots were used for each plant test: five treatments and three replicates per treatment. Five days after spraying, the treated plants were used for various tests.

#### 2.6.1. Relative Water Content (RWC)

Relative water content was calculated according to the following equation [26]:

RWC = (FW − DW)/FW) × 100

where FW is the fresh weight and DW is the dry weight of treated plants (dried to constant weight at 75 °C).

#### 2.6.2. Determination of Total Chlorophyll Content

Chlorophyll content was determined on fresh leaves five days after plant spraying. Here, 100 mg of leaves were ground in 5 mL acetone and kept for 72 h at 4 °C in the dark. The content of chlorophyll was determined according to the method of Lichtenthaler (1987), by measuring the absorbance at 663 and 646 nm [27].

#### 2.6.3. Relative Electrolyte Leakage

Relative electrolyte leakage was assessed in leaves of treated herbs to study the effect of *Eucalyptus* oils on solute leakage. Leaves were immersed in glass boxes containing distilled water for 60 min, and then the conductivity of the water solution was measured (C1). At this point, the aqueous solution was boiled for 30 min and its conductivity was measured (C2) [28]. The relative electrolyte leakage (REL) was calculated according to the following formula:

% REL = (C1/C2) × 100

#### 2.6.4. Determination of Free Proline Content

Proline levels were assessed according to the method adopted by (Bates et al., 1973) [29]. The fresh leaves of tested plants were digested with 6 mL of sulfosalicylic acid (3%) at 100 °C for 30 min, and then centrifuged at 25 °C at 2000× *g* for 5 min.

A volume of 0.5 mL of the obtained extract was mixed with 0.5 mL of distilled water and 2 mL of a mixture containing 0.5 g of ninhydrin, 30 mL of glacial acetic acid, and 20 mL of distilled water. The mixtures were boiled for 1 h. Then, the different samples were cooled and extracted with 6 mL of toluene. The toluene phase was used for absorbance measurements at 520 nm. The proline content was calculated from a standard curve. Proline content was expressed as mg/g fresh weight. All samples were analyzed in triplicate.

### 2.7. Antifungal Activity

Eight fungal strains (*Bipolaris sorokiniana*, *Fusarium verticillioides*, *F. pseudograminearum*, *F. proliferatum*, *F. nygamai*, *F. graminarium*, *F. avenaceum*, and *F. culmorum*) were obtained from the Laboratory of Plant Protection, Tunisian National Institute of Agronomic Research (INRAT). The antifungal potential of the *Eucalyptus* oils on the mycelial growth of tested fungi was assessed in vitro using the agar dilution method [16]. EOs tested were diluted in a solution (0.1%) of Tween 20 and then mixed in PDA medium to obtain a final concentration of 4 μL/mL. A disk of 5 mm, cut from the periphery of each fungal culture, was placed in the center of the PDA plate and then incubated for 7 days in optimal conditions. The experiments were carried out as three replicates per treatment. Growth inhibition was calculated as the growth percentage of inhibition relative to the control according to the formula:

% Inhibition ((C − T)/C) × 100

where C: mean of growth of three replicates (mm) of control plates. T: mean of growth of three replicates (mm) of plates containing *Eucalyptus* oils.

### 2.8. Statistical Analysis

All of the experiments were carried out in three replicates, with the results represented as mean ± standard deviation. Data were subjected to one-way analysis of variance (ANOVA) using the SPSS 18.0 software package. Differences between means were tested through Student–Newman–Keuls and values with *p* ≤ 0.05 were considered significantly different.

## 3. Results

### 3.1. Eucalyptus EOs’ Yield and Chemical Composition

The hydrodistillation of dried leaves yielded 0.7 ± 0.1%, 1.3 ± 0.1%, and 2.9 ± 0.2% of yellow oils for *E. falcata*, *E. sideroxylon*, and *E. citriodora*, respectively. Oils’ components, area percentages, retention indices, formulae, and the chemical classes of components for each oil are reported in Table 2.

**Figure 1:**
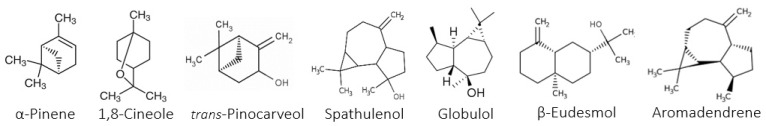
Major compounds of essential oils of *E. falcata*.

Analysis of the three oils allowed the identification of 58 compounds distributed across five classes of terpene and non-terpene derivatives. All oils showed a specific richness in oxygenated monoterpenes (51.3–90%) and oxygenated sesquiterpenes (4.8–29.4%). In *E. falcata*, 31 components were identified, representing 96.5% of the total EOs. Oxygenated monoterpenes and oxygenated sesquiterpenes were the two main subclasses in this oil (51.3% and 29.4%, respectively). EOs’ main constituents were 1,8-cineole (28.4%) and trans-pinocarveol (14.2%) as oxygenated monoterpenes, and globulol (9.1%) and spathulenol (7.2%) as oxygenated sesquiterpenes. Furthermore, appreciable percentages of the monoterpene hydrocarbon α-pinene (6.0%) were also present. The major and characteristic compounds of the essential oils of *E. falcata* are presented in Figure 1.

**Figure 2:**
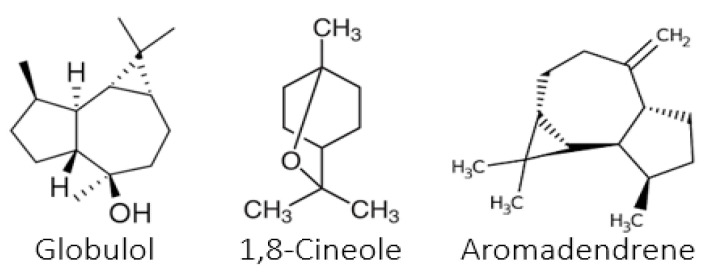
Major compounds of essential oils of *E. sideroxylon*.

In *E. sideroxylon* EOs, 33 components were identified, accounting for 99.3% of the oil. Oxygenated monoterpenes and oxygenated sesquiterpenes were the two main subclasses in this oil (75.5% and 16.2%, respectively). The EOs of this species were characterized by a high level of 1,8-cineole (65.4%) as the major oxygenated monoterpene and globulol (7.4%) as an oxygenated sesquiterpene. The major and characteristic compounds of the essential oils of this species are presented in Figure 2.

In *E. citriodora* EOs, 22 different chemical constituents were identified, accounting for 98.5% of the oil. Globally, *E. citriodora* EOs were characterized by the dominance of oxygenated monoterpenes (90%). The major components were citronellal (48.7%), citronellol (20.2%), aitronellyl acetate (8.1%), and isopulegol (8.1%).

**Figure 3:**
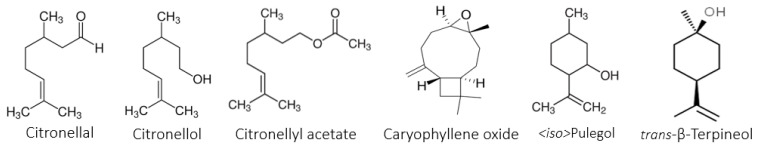
Major compounds of essential oils of *E. citriodora*.

Great diversity in the structure and nature of the compounds of *E. citriodora* EOs has been described. The chemical structure of the major compounds is shown in Figure 3.

### 3.2. Antioxidant Activity

To establish a better assessment of the antioxidant potential of the EOs, they were evaluated using three different methods. Table 3 details the results obtained in this study.

According to the statistical analysis, significant differences were noted in the TAC of the three tested EOs. The highest TAC value was observed for *E. citriodora* oil (80.21 mg GAE/g), followed by *E. falcata* oil (32.59 mg GAE/g) and *E. sideroxylon* (26.68 mg GAE/g) oils.

*E. citriodora* EOs showed also the highest antiradical activity with both the DPPH and ABTS assay (IC_50_ = 71.37 and 53.26 µg/mL, respectively).

Hence, the EOs of *E. citriodora* exhibited the highest antioxidant activity in all methods, while the lowest activity was obtained with the EOs of *E. falcata* and *E. sideroxylon*. The observed results could be related to the different compositions of the three oils.

### 3.3. Phytotoxic Effects of Eucalyptus EOs

#### 3.3.1. Anti-Germinative Activity

The phytotoxic effect of the *Eucalyptus* EOs was evaluated against two well-known weeds, *S. arvensis* and *P. canariensis*, and on one cultivated crop, *T. durum*. Germination and growth of all tested herbs decreased in a dose-dependent manner, as shown in Table 4.

Data showed that all oils significantly inhibited the seed germination of all tested herbs. The inhibition depended on both the tested species and dose. In fact, the inhibition of seed germination increased with increasing oil concentration.

A significant reduction in the seed germination of all tested herbs was observed at all doses. *E. falcata* oil caused complete germination inhibition in *S. arvensis* at 2 μL/mL. However, at the same dose, seed germination was only reduced for *P. canariensis* and *T. durum* (26.7 and 36.7%), respectively.

The volatile oil of *E. sideroxylon* reduced the germination of *S. arvensis* at the dose of 2.0 µL/mL to 16.6% and that of *P. canariensis* to 30%. On the contrary, *T. durum* appeared to be more resistant at the same dose, observing a higher percentage of germination (63.3%).

The high phytotoxicity of *E. citriodora* oil was remarkable, with a significant inhibitory effect against the germination of all tested herbs at all oil concentrations (0.5, 1.0, 1.5, and 2.0 µL/mL). Indeed, it was also remarkable that the complete inhibition of germination for *S. arvensis* by *E. citriodora* oil was obtained at the dose of 1 µL/mL. However, a partial reduction at the same dose (1.0 µL/mL) was observed in the germination of both *P. canariensis* and *T. durum*, although, at the highest dose (2.0 µL/mL), the seed germination of all tested herbs was totally inhibited.

Similarly, a high herbicidal effect was found with *Eucalyptus* oils against the seedling growth (roots and shoots) of all tested herbs at all doses; see Table 5 and Table 6.

The growth of *S. arvensis* seedlings was the most affected by *Eucalyptus* oil application, with high inhibition in both aerial parts and root growth when compared to the control at a low dose (0.5 µL/mL). Conversely, no significant effect at the same dose was observed for *P. canariensis* and *T. durum* (monocot species), which are more resistant to oil application.

#### 3.3.2. Post-Emergence Assays

**Figure 4:**
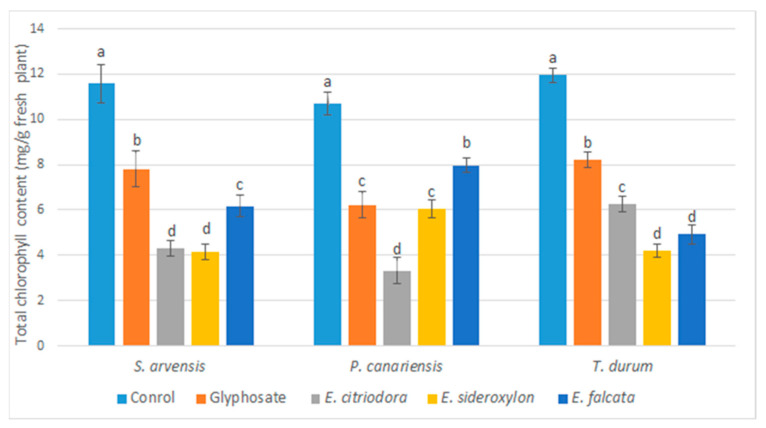
Effects of *Eucalyptus* species essential oils and glyphosate on total chlorophyll content as mg/g fresh tested plants measured five days after application. Error bars and letters on graphs represent standard error means and significant differences at *p* ≤ 0.05 through Student–Newman–Keuls.

The post-emergent spray treatment with *Eucalyptus* EOs and glyphosate caused chlorosis and necrosis of the leaves, breaking of the stem, and complete wilting of all the tested weeds (*P. canariensis* and *S. arvensis*) and the cultivated species (*T. durum*) when compared to the negative control. Five days after the application of the EOs, significant effects on chlorophyll synthesis were seen. In fact, a significant decrease in the chlorophyll rate was noted for all the tested herbs. This effect varied according to the applied EOs and the tested species (Figure 4).

Generally, all the oils showed similar and even better effects than glyphosate. For *S. arvensis* (dicot weed), a significant decrease in the synthesis of chlorophyll was noted in the order of 48.28, 61.74, and 63.37% for *E. falcata*, *E. citriodora*, and *E. sideroxylon*, respectively. On the contrary, glyphosate showed the lowest rate of inhibition (33.96%).

In the case of *P. canariensis* (monocot weed), all oils showed the significant inhibition of chlorophyll synthesis; *E. citriodora* showed the highest rate of inhibition (67.45%), followed by *E. falcata* (24.24%) and *E. sideroxylon* (44.87%), which had effects similar to glyphosate (42.61%).

For *T. durum*, significant inhibition was noted with all *Eucalyptus* oils and its effects outweighed the chemical herbicide used as a positive control. The inhibition of chlorophyll synthesis may explain the herbicidal potential of the *Eucalyptus* EOs and may explain the effects of chlorosis, necrosis, and stunted growth observed after their application.

**Figure 5:**
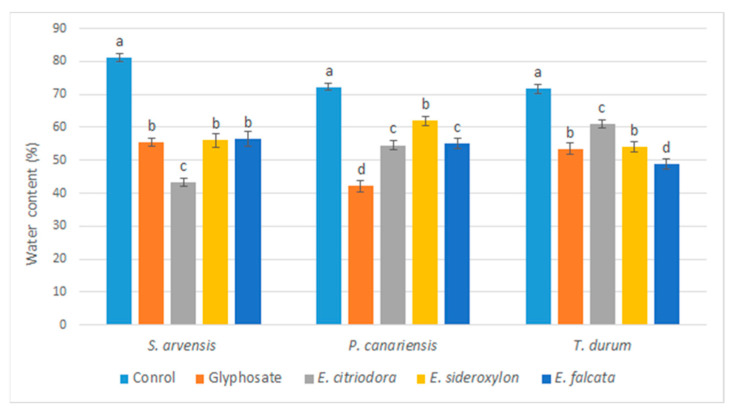
Effects of *Eucalyptus* essential oils and glyphosate on water content % of test plants measured five days after application. Error bars and letters on graphs represent standard error means and significant differences at *p* ≤ 0.05 through Student–Newman–Keuls.

In the second phase and in relation to the drying effect observed following the application of *Eucalyptus* oils, the relative water content of the treated herbs was determined. The obtained results are presented in Figure 5.

According to the statistical analysis, a significant and important decrease in the water content of the different tested herbs can be confirmed. This reduction varied according to the applied oil and the tested herb (mono- or dicotyledons and weeds or cultivated species). The effects observed are similar to those obtained with glyphosate. In fact, the water content was reduced for *S. arvensis* by 28.00, 29.26, and 46.4% by *E. falcata*, *E. sideroxylon*, and *E. citriodora*, respectively. For *P. canariensis*, the observed reductions were in the order of 24.6, 15.16, and 27.36%, respectively. In the case of *T. durum*, a smaller decrease in water content was observed with *E. citriodora* and *E. sideroxylon* (10.47 and 25.49%, respectively), while *E. falcata* caused the most significant decrease (49.57%), which outweighed the effects caused by glyphosate (27.5%).

The obtained results reflect the phytotoxic potential of *Eucalyptus* oils and can explain their mechanism of action, which consists in altering the water balance of the plant.

**Figure 6:**
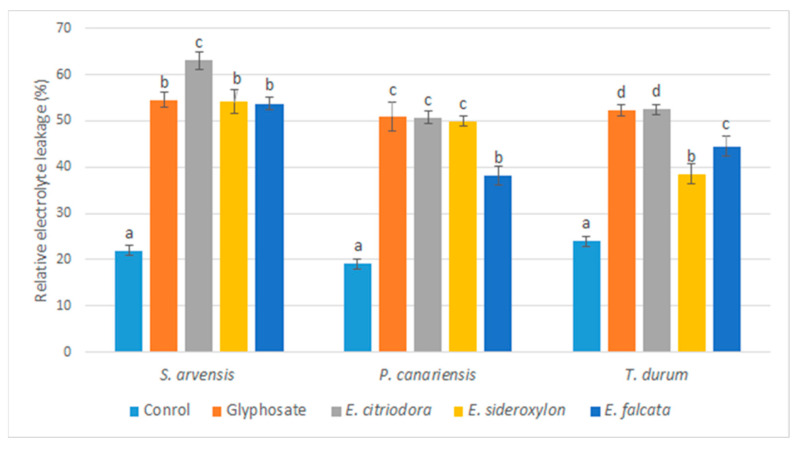
Effects of *Eucalyptus* essential oils and glyphosate on relative electrolyte leakage percentage of tested plants measured five days after spray. The errors bars and letters on graphs represent standard errors means and significant differences at *p* ≤ 0.05 through Student–Newman–Keuls.

In light of these observations, the effect of *Eucalyptus* oils on the membrane integrity of the tested herbs was evaluated by measuring the relative electrolyte leakage. It has been observed that exposure to *Eucalyptus* EOs induces significant electrolyte leakage from the leaves of the tested herbs, as assessed by the increase in the conductivity of the bathing medium. Hence, these volatile oils disrupt the membrane integrity and cause solute leakage (Figure 6).

According to the statistical analysis, very pronounced relative electrolyte leakage was noted following the application of EOs and glyphosate compared to the control.

Electrolyte leakage explains the loss of plants’ membrane integrity and therefore the observed phytotoxic effects.

The effect of the three oils on electrolyte leakage varied according to the species of *Eucalyptus* and that of the tested plants. Volatile oils of *E. citriodora* induced the most pronounced effects on all herbs: 54.06, 55.23, and 66.93% for *T. durum*, *P. canariensis*, and *S. arvensis*, respectively.

Again, the dicot weed *S. arvensis* was the most sensitive to the action of all applied oils.

**Figure 7:**
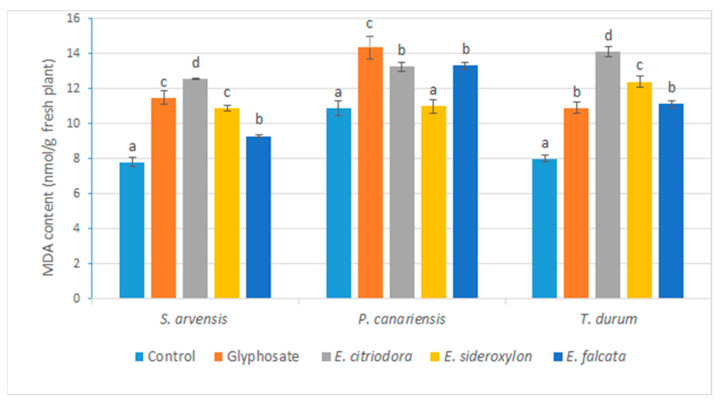
Effects of *Eucalyptus* species essential oils and glyphosate on MDA content as nmol/g fresh tested plants measured five days after application. Error bars and letters on graphs represent standard error means and significant differences at *p* ≤ 0.05 through Student–Newman–Keuls.

The lipid peroxidation of polyunsaturated fatty acids, of which malondialdehyde is a product, could also provide an explanation for the relative leakage of electrolytes and the loss of membrane integrity. For this reason, we measured the level of MDA in the aerial parts of tested species after the application of the oils and the positive and negative controls. The main results are shown in Figure 7.

Based on the statistical analysis of the obtained data, an increase in the MDA level was noted for all oils and for glyphosate compared to the control. The highest MDA levels for all the tested herbs were obtained with *E. citrioodora* oil and glyphosate. These results confirm and can be correlated with the loss of membrane integrity and confirm the phytotoxicity of EO application.

It is also known that allelopathic substances generate oxidative stress in plants. This stress is particularly reflected in the accumulation of proline. In this context, the proline level in the aerial parts of the plants sprayed with the three *Eucalyptus* oils was measured.

**Figure 8:**
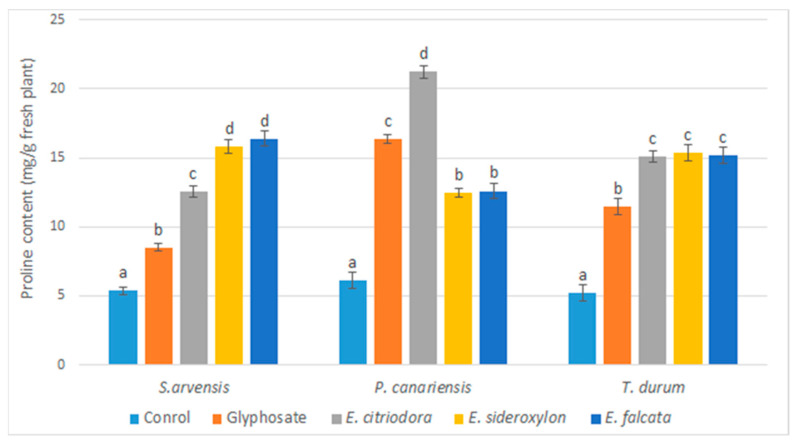
Effects of *Eucalyptus* essential oils and glyphosate on proline content as mg/g fresh tested plants measured five days after application. Error bars and letters on graphs represent standard error means and significant differences at *p* ≤ 0.05 through Student–Newman–Keuls.

The results obtained are summarized in Figure 8, which shows an increase in proline levels in the aerial parts of sprayed weeds and crops.

This accumulation varied according to the oils tested and the herbs used for phytotoxicity tests. Generally, EOs showed similar effects to the commercial herbicide. The effect of *E. citriodora* oil was most pronounced for *P. canariensis* (22.36 mg/g f.w.), three times greater than the control (6.39 mg/g f.w.), and *S. arvensis* (12.41 mg/g f.w.), more than two times greater than the control (5.36 mg/g f.w.). In the case of *T. durum*, all the oils showed a similar effect on the accumulation of proline.

To evaluate the possible antifungal activity, the three *Eucalyptus* EOs were tested against eight phytopathogenic fungi that attack cereals and fruits; the main results are presented in Table 7.

The data in Table 7 show that the oils, at the dose of 4 μL/mL, significantly inhibited the growth of all tested fungi. According to the statistical analysis, the oils exhibited varying degrees of inhibition. *B. sorokiniana* was the most resistant to all the oils, while *Fusarium* species were the most sensitive. *E. citriodora* EOs showed the best antifungal properties, causing total inhibition for all strains, except for *B. sorokiniana*. The other two EOs caused partial inhibition on nearly all tested strains.

## 4. Discussion

The study of the chemical composition of the essential oils of the three species of *Eucalyptus* revealed the great importance and richness of various compounds. According to the literature, among the few studies on *E. falcata* EOs, three studies were performed on plant material from Tunisia [17,18,19]. The chemical composition of the EOs of *E. falcata* is in agreement with that reported in the present study. In fact, the authors stated that the major components were 1,8-cineole (30.7%), trans-pinocarveol (26%), globulol (7%), and α-pinene (6%) [17]. However, the main differences with the previous studies consist in the absence of spathunelol, the presence of globulol, and the high levels of trans-pinocarveol [17,18].

Similar results were obtained in previous reports for *E. sideroxylon* [15,19]. According to these studies, the EOs of *E. sideroxylon* were characterized by their high content of 1,8-cineole (69–81%), which is in agreement with the present study.

Several investigations on *E. citriodora* EOs have been reported worldwide. In fact, in Colombian *E. citriodora* oils [30,31], in India [20], in Taiwan [32], and in the Congo [33], the major components were citronellal (40–72%), citronellol (6–22%), and iso-pulegol (3–13%), which is in agreement with our obtained data. However, citronellyl acetate was detected for the first time in this study. On the other hand, our study is in contrast with the results of a Tunisian report [2,34] wherein the authors evidenced a different chemotype characterized by a specific richness in 1,8-cineole (54%) and α-pinene (23%) and the total absence of citronellal, citronellol, and iso-pulegone. Apart from this, the data of the current study are in good agreement with the literature. The small differences in the percentages may be due to several factors, such as the genetic background, biotic and abiotic factors, season of collection, and extraction methods [35].

The evaluation of the antioxidant potential of the three oils showed interesting activities that can contribute to the valorization of essential oils of forest species, particularly of the *Eucalyptus* genus. These results are in agreement with the literature.

Indeed, several studies have investigated the antioxidant properties of different *Eucalyptus* species, such as *E. oleosa*, *E. grandis* × *E. urophylla*, *E. gracilis*, *E. citriodora*, *E. salubris*, and *E. salmonophloia* [36,37,38,39].

Furthermore, the EOs of *E. citriodora* exhibited higher antioxidant potential than those of *E. falcata* and *E. sideroxylon*. This appears to be related to the high level of oxygenated terpenes in *E. citriodora* oil [36,37].

Similarly, the EOs of *E. citriodora*, collected in India, contain oxygenated monoterpenes such as citronellal (60.66%), exhibiting strong antioxidant activity, in agreement with the current study [38]. In the same report, the authors evaluated and showed the good antioxidant activity of α-citronellal, β-citronellol, and isopulegol, the major compounds of the oil. Furthermore, the synergism between oil components could be related to the remarkable antioxidant capacity of the tested oils [40].

The study of the allelopathic properties of the EOs obtained from the three species of *Eucalyptus* revealed remarkable herbicidal potential, which even exceeds the activity of the synthetic herbicide glyphosate.

The germination- and growth-inhibiting effects of the *Eucalyptus* oils tested in the present study appear to be linked to the presence of several chemical components, particularly monoterpenes and sesquiterpenes, which are the main constituents and are known for their herbicidal potential [41]. Indeed, EOs of species belonging to the *Myrtaceae* family are known for their richness in allelochemicals [5,8,9,16,42]. In particular, in previous studies, we showed the phytotoxic potential of volatile oils and crude extracts of *Eucalyptus erythrocorys* against weeds [16,42].

Recently, the herbicidal potential of 22 *Eucalyptus* species growing in Tunisia has been reported. These studies reveal enormous allelopathic potential linked to the EOs of *Eucalyptus* species; this could be in agreement with the present study [5,8,9,16].

Observing the chemical composition of the *Eucalyptus* oils tested in the present work (Table 2), we can assume that the activity is due to different components, such as 1,8-cineole (0.3–65.4% in *Eucalyptus* oils), citronellal (48.7% in *E. citriodora* oil), citronellol (20.2% in *E. citriodora* oil), and α-pinene (ranging between 0 and 6% in *Eucalyptus* oils), already known for their significant phytotoxic effects [20,43,44].

Several studies report that terpenes, both hydrocarbons and oxygenated, are phytotoxic molecules. Additionally, oxygenated monoterpenes (51.3, 75.5, and 90%, respectively, in *E. falcata*, *E. sideroxylon*, and *E. citriodora*) have been reported to have stronger herbicidal properties than the corresponding hydrocarbon derivatives [20,43,44]; this can explain the high herbicidal activity of *E. citriodora* compared to *E. falcata* and *E. sideroxylon*.

To explain the mode of action of terpenes on the inhibition of germination, various physiological and biochemical mechanisms have been reported.

In fact, such monoterpenes can affect physiological functions, such as cell division, viability, and growth inhibition by the alteration of membrane integrity [45,46].

Other studies have reported that EOs reduce germination and growth by inhibiting the incorporation of nitrogen into amino acid synthesis, leading to the accumulation of ammonia, and by impairing photosynthesis and photorespiration [47].

In the same way, Abrahim et al., 2003 demonstrated that the monoterpene hydrocarbon α-pinene reduces the growth of maize by inhibiting electron transfer and uncoupling oxidative phosphorylation, resulting in the alteration of energy metabolism and the blocking of ATP synthesis in the mitochondria [48]. Similarly, some sesquiterpenes have been reported to have phytotoxic effects by causing oxidative stress, the inhibition of photosynthesis, and the induction of microtubular alterations [47]. Previous reports have shown that EOs induce oxidative stress to weeds and increase peroxidase and superoxide dismutase activities [49,50,51]. Other studies have shown that EOs and their constituents have remarkable herbicidal potential, the effects of which result in anatomical and physiological effects, such as the formation of lipid globules in the cytoplasm, inhibition of mitochondrial development, and alteration of the integrity of the membrane of the nucleus and mitochondria [52,53].

For post-emergence trials and the application of EOs by spraying, few studies have been undertaken and the exact modes of action still remain poorly known. On the other hand, data obtained in this study are in agreement with those reported in the literature. Several studies have reported that various constituents of EOs, when applied at the post-emergence stage, are able to inhibit the growth of weeds [54], hypothesizing oxidative stress and alteration of the water content [47]. The evidence that emerged in the present study showed a correlation between the decrease in the water content and the arrest of the growth of the plants treated with *Eucalyptus* EOs.

Following the application of *Eucalyptus* EOs, a marked decrease in chlorophyll synthesis was noted, suggesting a correlation between the visible effects on weeds (i.e., chlorosis, necrosis, and photosynthetic functions) and their growth. According to previous studies, several EOs and their pure components have been described to inhibit photosynthesis and chlorophyll synthesis [55]. Indeed, some oxygenated monoterpenes have been reported to induce a photosynthetic decrease by reducing the chlorophyll content in various weeds [56].

In addition, citronellol and 1,8-cineole, major compounds of the *Eucalyptus* species in the current study, reduced the chlorophyll content of *Ageratum conyzoides* by 60 and 66%, respectively [57,58]. Earlier studies concluded that the decrease in chlorophyll content appeared to be related to the inhibition of chlorophyll synthesis and degradation of photosynthetic pigments [59,60].

Among the physiological effects of *Eucalyptus* EOs on membrane integrity, the excessive leakage of electrolytes was noted, which can be explained by the loss of integrity of membrane cells. On the other hand, membrane integrity is essential for vital functions and plays several roles, including that of a barrier, selective permeability to nutrients, and, indeed, control of the electrolyte balance [61]. Various secondary metabolites have been reported to alter the integrity of the cell membrane in plants, particularly EOs and their pure constituents. A previous study has shown that linalool caused increased membrane permeability, further demonstrating that EOs can cause important damage to membrane permeability [62]. According to the data obtained, the application of *Eucalyptus* EOs induced, in the tested herbs, an increase in the rate of MDA and proline compared to the control, in agreement with the previous observations. Indeed, it has been reported that the application of EOs during the post-emergence stage increases the permeability of the membrane by disrupting its integrity because of lipid peroxidation [47,63,64,65]. Additionally, some EOs and monoterpenes have been reported to induce oxidative stress. α-pinene caused lipid peroxidation when applied to *Cassia occidentalis*, resulting in increased solute leakage [25]. Furthermore, the increase in MDA levels was related to the lipid peroxidation of the membrane and alteration of its permeability.

Finally, the current study has shown that *Eucalyptus* oils have significant in vitro antifungal potential against the growth of phytopathogenic fungi.

The differences in the resistance of different fungal strains to *Eucalyptus* volatile oil seem to be related to the different capacities of the oil components to disrupt the cell membrane [66].

*Eucalyptus* oils are known to have remarkable antimicrobial activity that is related to their richness in oxygenated compounds [67].

Indeed, we have previously reported the antifungal potential of *Eucalyptus erhytrocorys* EOs and its crude extracts against phytopathogenic fungi [16,42], which is in agreement with the current study. Similarly, the inhibitory activity of the volatile oil obtained from *Eucalyptus camaldulensis* against the growth of fungi such as *Thanatephorus cucumeris*, *Aspergillus niger*, *F. oxysporum*, and *Rhizopus oryzae* is also known [67].

In agreement with the current study, Ivanov et al. (2021) demonstrated the fungicidal activity of 1,8-cineole and camphor (major compounds in *Eucalyptus* volatile oils) against the growth of different *Candida* species [68]. In another research work, it was reported that terpinen-4-ol and α-terpineol, terpinolene, and 1,8-cineole, constituents of *Eucalyptus* oils, are fungitoxic against *Botrytis cinerea* [69]. However, with regard to the antimicrobial mechanism, it has been reported that the apolar nature of terpenes may favor their penetration into the lipid bilayer of the fungal membrane, inducing its disruption. Many terpenes have been reported to exert their antifungal activity by increasing the permeability of fungal cells and membrane fluidity [70].

## 5. Conclusions

To conclude, our data showed that *Eucalyptus* EOs are a complex mixture of various compounds. The chemical profile of the tested EOs was characterized by the high content of oxygenated monoterpenes. Great diversity was noted between the three species with the identification of different major compounds; however, the three species belong to the same genus. This is an interspecific diversity, with the identification of 58 compounds. Similarly, the variability of the results obtained in this present study with those in the literature could be considered within the context of ecological variability, which makes this work necessary to appreciate the results obtained with these three species of *Eucalyptus* growing in Tunisia. The essential oils tested for their biological activities have shown efficacy for the control of weeds and phyto-pathogenic fungi, and their antioxidant potential has also been described. These biological activities offer several areas of application for these oils, whether in agronomy or in the chemical and pharmaceutical industries. These molecules are of natural identity; they are biodegradable molecules and are compatible with the environment. Their applications could solve several problems, including the problem of resistance and the undesirable environmental effects. Plants synthesize these substances as allelochemicals; their exploitation can provide an enhancement in forest species and also allow the exploitation of the allelopathic potential of aromatic and medicinal plants. Consequently, the adoption of such bio-molecules as novel compounds in herbicidal formulations could facilitate not only sustainable weed control but also sustainable agriculture. Further studies are required to evaluate their application under field conditions. Such results encourage the use of *Eucalyptus* EOs as natural products in agriculture and for their agro-industrial potential.

## Figures and Tables

**Table 1:** Species, date and site of harvest of the plant material.

Species	Part Collected	Date of Harvest	Harvest Site	Preserved Specimen	Bioclimatic Stage
*E. citriodora*	Leaves	December 2020	Rimel arboreta, Bizerte	EC2026	Humid inferior with mild winter
*Eucalyptus sideroxylon*	Leaves	October 2020	Henchir En Naâm arboreta, Siliana	ES2027	Upper semi-arid
*E. falcata*	Leaves	October 2020	Mjez Elbab arboreta, Beja	EF2028	Upper semi-arid with mild winter
*S. arvensis*	Seeds	July 2019	Sidi Ismail, Beja	SA1948	Subhumid with mild winter
*P. canariensis*	Seeds			SA1949	
*T. durum*	Seeds			SA1952	

**Table 2:** Chemical compositions of essential oils of *Eucalyptus* species.

Peak	Component	Formula	LRI (Lit.)	LRI	%		
					*E. falcata*	*E. sideroxylon*	*E. citriodora*
1	α-pinene	C_10_H_16_	939	941	6	1	
2	*p*-cymene	C_10_H_14_	1026	1028	2.5	0.9	
3	limonene	C_10_H_16_	1031	1032	0.7	0.9	0.1
4	1,8-cineole	C_10_H_18_O	1033	1034	28.4	65.4	0.3
5	bergamal	C_11_H_18_O	1053	1053			0.3
6	isopentyl isovalerate	C_10_H_20_O	1103	1104		0.1	
7	*cis*-rose oxide	C_11_H_18_O	1111	1111			0.1
8	fenchol	C_10_H_18_O	1110	1112	0.7		
9	α-campholenal	C_10_H_16_O	1124	1126	0.2		
10	*trans*-rose oxide	C_11_H_18_O	1126	1127			0.2
11	*trans*-pinocarveol	C_10_H_16_O	1139	1141	14.2	1.8	
12	*iso*pulegol	C_10_H_18_O	1146	1146			8.1
13	citronellal	C_10_H_18_O	1153	1155			48.7
14	*trans*-β-terpineol	C_10_H_18_O	1162	1160			4.1
15	pinocarvone	C_10_H_14_O	1162	1164	2.2	0.2	
16	borneol	C_10_H_18_O	1165	1166	1.1	0.2	
17	*trans*-ocimenol	C_10_H_16_O	1168	1169		0.2	
18	4-terpineol	C_10_H_18_O	1177	1179	0.6	0.4	
19	*p*-cymen-8-ol	C_10_H_14_O	1183	1184	0.4	0.3	
20	*p*-mentha-1(7),8-dien-2-ol	C_10_H_16_O	1186	1186	0.7	1.5	
21	α-terpineol	C_10_H_18_O	1189	1191	1.5	1.4	
22	myrtenol	C_10_H_16_O	1191	1193	0.4		
23	*trans*-carveol	C_10_H_16_O	1217	1220	0.3	0.3	
24	*cis*-carveol	C_10_H_16_O	1229	1228		1.6	
25	citronellol	C_10_H_20_O	1228	1230			20.2
26	*(Z)*-tagetone	C_10_H_16_O	1234	1231		0.9	
27	carvone	C_10_H_14_O	1242	1244		0.2	
28	geraniol	C_10_H_18_O	1255	1256			0.1
29	citronellyl formate	C_11_H_20_O_2_	1275	1276			0.1
30	carvacrol	C_10_H_14_O	1298	1298	0.4		
31	*p*-vinylguaiacol	C_9_H_10_O_2_	1312	1314			0.4
32	3,7-dimethyl-6-octenoic acid	C_10_H_18_O_2_	1314	1315			1.2
33	2-acetoxy-1,8-cineole	C_12_H_20_O_3_	1345	1345	0.2	0.2	
34	α-terpinyl acetate	C_12_H_20_O_2_	1350	1352		0.9	
35	citronellyl acetate	C_12_H_22_O_2_	1354	1354			8.1
36	2-phenylethyl isobutyrate	C_12_H_16_O_2_	1395	1394			0.1
37	*(Z)*-jasmone	C_11_H_16_O	1394	1395			0.4
38	β-caryophyllene	C_15_H_24_	1418	1419	0.6	0.3	1
39	isoamyl benzoate	C_12_H_16_O_2_	1439	1439			
40	aromadendrene	C_15_H_24_	1440	1440	3.2	2.1	
41	α-humulene	C_15_H_24_	1454	1455			0.2
42	alloaromadendrene	C_15_H_24_	1460	1462	0.8	0.6	
43	β-selinene	C_15_H_24_	1486	1487	0.4		
44	α-vetispirene	C_15_H_22_	1483	1488	0.5		
45	β-phenylethyl isovalerate	C_13_H_18_O_2_	1489	1490		0.3	
46	α-selinene	C_15_H_24_	1497	1495		0.2	
47	bicyclogermacrene	C_15_H_24_	1496	1496	1.1	0.2	
48	germacrene B	C_15_H_24_	1556	1557		1	
49	ledol	C_15_H_26_O	1565	1566	1	0.7	
50	spathulenol	C_15_H_24_O	1576	1576	7.2	3.3	1.4
51	caryophyllene oxide	C_15_H_24_O	1582	1582			2.5
52	globulol	C_15_H_26_O	1584	1583	9.1	7.4	
53	viridiflorol	C_15_H_26_O	1590	1591	3.6	1.6	
54	rosifoliol	C_15_H_26_O	1599	1601	1.5	0.6	
55	10-*epi*-α-eudesmol	C_15_H_26_O	1617	1620	1.9	0.6	0.5
56	*iso* spathulenol	C_15_H_24_O	1640	1640	0.7		
57	β-eudesmol	C_15_H_26_O	1649	1650	4.4	2	
58	α-cadinol	C_15_H_26_O	1653	1652			0.4
Monoterpene hydrocarbons %					9.2	2.8	0.1
Oxygenated monoterpenes %					51.3	75.5	90
Sesquiterpene hydrocarbons %					6.6	4.4	1.2
Oxygenated sesquiterpenes %					29.4	16.2	4.8
Non-terpene derivatives %					0	0.4	2.4
Total identified %					96.5	99.3	98.5

**Table 3:** Antioxidant activity of the essential oils of the three *Eucalyptus* species.

Essential Oils	TAC (mg GAE/g)	DPPH (IC_50_ μg/mL)	ABTS (IC_50_ μg/mL)
*E. citriodora*	80.21 ± 5.72 a	71.37 ± 3.89 b	53.26 ± 5.57 b
*E. sideroxylon*	26.68 ± 6.25 b	96.29 ± 5.58 c	84.84 ± 1.52 c
*E. falcata*	32.59 ± 3.83 b	112.61 ± 3.86 d	90.35 ± 3.27 c
BHT	-	20.53 ± 2.79 a	-
Trolox	-	-	15.75 ± 1.11 a

TAC: total antioxidant capacity; DPPH: 2,2-diphenyl-1-picrylhydrazyl; ABTS: 2,2′-Azino-bis(3-ethylbenzthiazoline-6-sulfonic acid; GAE: gallic acid equivalent; BHT: butylated hydroxytoluene. Results are expressed as mean ± standard deviation of 3 repetitions. Different letters in the same column indicate significant differences (*p* ≤ 0.05) through Student–Newman–Keuls.

**Table 4:** Inhibitory effect of *Eucalyptus* species EOs on seed germination.

Tested Plant	Dose (µL/mL)	Germination %			
		*E. falcata*	*E. sideroxylon*	*E. citriodora*	Glyphosate
*S. arvensis*	0	100 ± 0.0 a	100 ± 0.0 a	100 ± 0.0 a	100 ± 0.0 a
	0.5	46.6 ± 11.54 bC	76.6 ± 5.7 bB	33.3 ± 5.8 bC	96.66 ± 3.33 aA
	1	23.3 ± 5.8 cB	40 ± 10 cA	6.7 ± 5.8 cC	90 ± 5.77 aA
	1.5	16.7 ± 5.8 cB	23.3 ± 5.7 dB	0 ± 0 cC	86.66 ± 3.33 aA
	2	0 ± 0 dC	16.6 ± 5.7 dB	0 ± 0 cC	73.33 ± 3.33 bA
*P. canariensis*	0	90 ± 10 a	90 ± 10 a	90 ± 10 a	90 ± 10 a
	0.5	76.7 ± 5.8 aB	90 ± 10 aA	66.7 ± 5.8 bB	93.33 ± 3.33 aA
	1	46.7 ± 11.5 bC	100 ± 00 aA	43.3 ± 5.8 cC	80 ± 0.0 aB
	1.5	23.3 ± 5.8 cB	80 ± 10 aA	16.7 ± 5.8 dB	80 ± 0.0 aA
	2	26.7 ± 5.8 cB	30 ± 10 bB	3.3 ± 5.8 eC	66.66 ± 3.33 bA
*T. durum*	0	96.7 ± 5.7 a	96.7 ± 5.7 a	96.7 ± 5.7 a	96.7 ± 5.7 a
	0.5	86.7 ± 5.8 aB	83.3 ± 5.7 aB	76.7 ± 5.8 bB	100 ± 0.0 aA
	1	63.3 ± 5.8 bB	96.6 ± 5.7 aA	40 ± 10 cC	93.33 ± 3.33 aA
	1.5	30 ± 10 cB	90 ± 10 aA	13.3 ± 5.8 dC	80 ± 0.0 bA
	2	36.7 ± 5.8 cC	63.3 ± 10 bB	0 ± 0 eD	76.66 ± 3.33 bA

Values are means ± standard errors (*n* = 3). The lowercase letters compare, in the columns, the dose effect for each herb vis-à-vis each oil. The capital letters compare, in the rows, the sensitivity of each herb at the same dose vis-à-vis the samples tested. Means followed by the same letter are not significantly different by the Student–Newman–Keuls test (*p* ≤ 0.05).

**Table 5:** Inhibitory effect of *Eucalyptus* species essential oils on aerial parts growth of germinated seeds.

Tested Plant	Dose (µL/mL)	Shoots (cm)			
		*E. falcata*	*E. sideroxylon*	*E. citriodora*	Glyphosate
*S. arvensis*	0	7.7 ± 0.68 a	7.7 ± 0.68 a	7.7 ± 0.68 a	7.7 ± 0.68 a
	0.5	6.36 ± 1.32 aA	6.3 ± 0.58 bA	1.83 ± 0.74 bB	5.66 ± 0.28 bA
	1	4.1 ± 0.96 bA	4.7 ± 0.37 cA	1.13 ± 1.06 bcB	5.96 ± 0.38 bA
	1.5	2.93 ± 0.49 bA	2.73 ± 0.5 dA	0 ± 0 cB	3.06 ± 0.58 cA
	2	0 ± 0 cC	2.43 ± 0.49 dA	0 ± 0 cC	1.33 ± 0.17 dB
*P. canariensis*	0	9.76 ± 0.5 a	9.76 ± 0.5 a	9.76 ± 0.5 a	9.76 ± 0.5 a
	0.5	8.4 ± 2.16 abA	11.8 ± 1.58 aA	10.53 ± 0.61 aA	8.46 ± 0.41 bA
	1	9.13 ± 0.3 aA	8.8 ± 0.96 aA	7.5 ± 1.04 bA	4.56 ± 0.34 cB
	1.5	6.06 ± 0.9 bA	6.5 ± 2.02 abA	6.1 ± 0.75 cA	2.23 ± 0.33 dB
	2	3.3 ± 1.6 cA	3.4 ± 4.2 bA	0.4 ± 0.7 dA	0.66 ± 0.16 eA
*T. durum*	0	9.16 ± 1.2 a	9.16 ± 1.2 b	9.16 ± 1.2 a	9.16 ± 1.2 a
	0.5	8.73 ± 1.27 aB	11.53 ± 0.98 aA	8.56 ± 0.45 aB	5.36 ± 1.12 bC
	1	7.73 ± 0.76 aB	10.1 ± 1.4 abA	6.1 ± 0.2 bB	3.63 ± 0.18 bcC
	1.5	5.56 ± 0.81 bB	8.2 ± 0.36 bcA	4.56 ± 1.15 cB	2.2 ± 0.17 cdC
	2	3.6 ± 1.04 cB	6.5 ± 0.45 cA	0 ± 0 dC	0.9 ± 0.15 dC

Values are means ± standard errors (*n* = 3). The lowercase letters compare, in the columns, the dose effect for each herb vis-à-vis each oil. The capital letters compare, in the rows, the sensitivity of each herb at the same dose vis-à-vis the samples tested. Means followed by the same letter are not significantly different by the Student–Newman–Keuls test (*p* ≤ 0.05).

**Table 6:** Inhibitory effect of *Eucalyptus* species essential oils on roots growth of germinated seeds.

Tested Plant	Dose (µL/mL)	Roots (cm)			
		*E. falcata*	*E. sideroxylon*	*E. citriodora*	Glyphosate
*S. arvensis*	0	11.73 ± 0.7 a	11.73 ± 0.7 a	11.73 ± 0.7 a	11.73 ± 0.7 a
	0.5	10.07 ± 1.4 bA	7.8 ± 0.45 bB	3.77 ± 0.76 bD	5.86 ± 0.24 bC
	1	8.2 ± 0.5 cA	7.03 ± 1.4 bA	1.5 ± 1.32 cB	5.8 ± 0.55 bA
	1.5	5.7 ± 0.6 dA	4.6 ± 0.7 cB	0 ± 0 cD	2.36 ± 0.31 cC
	2	0 ± 0 eC	3.87 ± 0.55 cA	0 ± 0 cC	1.73 ± 0.21 cB
*P. canariensis*	0	10.3 ± 1.18 a	10.3 ± 1.18 a	10.3 ± 1.18 a	10.3 ± 1.18 a
	0.5	7.8 ± 0.4 abC	9.93 ± 1.33 aBC	11.2 ± 1.31 aA	9.66 ± 0.43 aBC
	1	8.7 ± 1.5 abB	9.46 ± 0.75 aA	7.5 ± 0.47 bBC	6.2 ± 0.25 bC
	1.5	6.5 ± 1.3 bcA	4.5 ± 1.23 bA	6.06 ± 1.0 bA	2.36 ± 0.32 cB
	2	4.8 ± 1.2 cA	2.33 ± 0.85 cB	0.4 ± 0.7 cC	0.63 ± 0.18 dC
*T. durum*	0	10.2 ± 1.09 a	10.2 ± 1.09 a	10.2 ± 1.09 a	10.2 ± 1.1 a
	0.5	10.4 ± 0.6 aA	7.4 ± 1.11 bB	9.06 ± 1.55 aAB	8.2 ± 0.5 bAB
	1	8.43 ± 0.45 aA	6.5 ± 0.7 bB	5.87 ± 0.76 bB	3.0 ± 0.15 cC
	1.5	5.83 ± 1.51 bA	5.7 ± 0.76 bA	5.73 ± 0.38 bA	2.23 ± 0.14 cdB
	2	3.9 ± 1.02 cB	7.36 ± 0.7 bA	0 ± 0 cD	1.2 ± 0.15 dC

Values are means ± standard errors (*n* = 3). The lowercase letters compare, in the columns, the dose effect for each herb vis-à-vis each oil. The capital letters compare, in the rows, the sensitivity of each herb at the same dose vis-à-vis the samples tested. Means followed by the same letter are not significantly different by the Student–Newman–Keuls test (*p* ≤ 0.05).

**Table 7:** Antifungal activity of *E. citriodora*, *E. sideroxylon*, and *E. falcata* essential oils.

Fungal Strain	Essential Oil Inhibition % at the Dose of 4 µL/mL		
	*E. citriodora*	*E. sideroxylon*	*E. falcata*
*F. culmorum*	100 ± 0.0 a	80.01 ± 7.51 b	100 ± 0.0 a
*F. pseudograminearum*	100 ± 0.0 a	70.49 ± 4.73 b	100 ± 0.0 a
*F. graminearum*	100 ± 0.0 a	80 ± 6.65 b	81.59 ± 4.27 b
*F. proliferatum*	100 ± 0.0 a	51.07 ± 6.96 b	87.06 ± 7.41 a
*F. avenaceum*	100 ± 0.0 a	62.55 ± 5.01 c	74.77 ± 2.39 b
*F.* *nygamai*	100 ± 0.0 a	74.55 ± 1.11 c	90.00 ± 4.71 b
*F. verticillioides*	100 ± 0.0 a	78.11 ± 5.23 b	73.52 ± 4.15 b
*B. sorokiniana*	77.64 ± 6.65 a	48.70 ± 8.26 a	66.66 ± 11.78 a

Results are expressed as mean ± standard errors. Different letters in the same row indicate significant differences (*p* ≤ 0.05).

## Data Availability

All data are available in the manuscript file.
